# Kidney biopsy in the diagnosis of immunoglobulin A nephropathy

**DOI:** 10.3389/fmed.2026.1882638

**Published:** 2026-10-07

**Authors:** Kirk N. Campbell, Anthony Chang, Marina Vivarelli

**Affiliations:** 1 Division of Renal-Electrolyte and Hypertension, University of Pennsylvania Perelman School of Medicine, Philadelphia, PA, United States; 2 Department of Pathology, The University of Chicago, Chicago, IL, United States; 3 Clinical Trial Center and Laboratory of Nephrology, Bambino Gesù Children's Hospital IRCCS, Rome, Italy

**Keywords:** complement, diagnosis, glomerulonephritis, IgAN, kidney biopsy

## Abstract

Immunoglobulin A nephropathy (IgAN) is a kidney disease characterized by deposition of galactose-deficient IgA1-containing immune complexes in the glomerular mesangium. It is the most common primary glomerular disease worldwide, affecting approximately 2.5 persons per 100,000 population annually. A definitive IgAN diagnosis requires a kidney biopsy. However, non-specific clinical signs, limited disease awareness, and lack of validated biomarkers can delay nephrology referral from primary care providers, prolonging time to diagnosis and negatively impacting patient outcomes. In this perspective, we aim to raise awareness of the importance of timely kidney biopsy in IgAN. To do so, we first outline the key clinical features of IgAN and kidney biopsy indications that should prompt nephrology referral. Subsequently, we highlight the diagnostic and prognostic significance of kidney biopsy findings, examine barriers to kidney biopsy completion, and provide our outlook on the ways in which kidney biopsy may be useful for IgAN management in the future.

## 1 Introduction

Immunoglobulin A nephropathy (IgAN) is the most common primary glomerular disease worldwide, characterized by the deposition of galactose-deficient IgA1-containing immune complexes in the glomerular mesangium (1). Mesangial deposition of these immune complexes activates multiple downstream pathways, including the complement and endothelin systems, leading to tissue injury and kidney damage (1, 2). IgAN contributes significantly to the global burden of chronic kidney disease (CKD) (1, 3), with up to 50% of patients progressing to end-stage kidney disease (ESKD) within 10–20 years of diagnosis (4–6). Globally, the annual incidence of IgAN in adults is estimated at 2.5 per 100,000 population, varying from 0.2 to 5.0 cases per 100,000 (7), while the reported range in children varies from 0.1 to 9.9 cases per 100,000 (8).

Healthcare providers may suspect IgAN in some patients following clinical presentation and/or laboratory testing (9). However, a definitive IgAN diagnosis can only be made following a kidney biopsy showing predominant or co-dominant glomerular IgA deposits, which is the defining pathologic criterion for the disease (10). Concerningly, a recently published, real-world, multinational, retrospective analysis showed that 14% of patients (251/1,792) with a diagnosis of IgAN had not undergone a kidney biopsy (9). Kidney biopsy is essential not only to exclude alternative diagnoses and establish the correct diagnosis, but to assess the extent of disease severity, inflammation, and scarring, and to maximize patient outcomes. Therefore, in this perspective, we aim to raise awareness of the importance of timely kidney biopsy in the diagnosis and management of IgAN as a means to improve outcomes in this patient population. To do this, we review recommendations for the diagnosis of IgAN by kidney biopsy and discuss the key clinical features that should prompt nephrology referral for kidney biopsy and further evaluation. In addition, we highlight the diagnostic and prognostic significance of kidney biopsy in IgAN, examine existing barriers to its use, and provide our outlook on the potential utility of biopsy in the rapidly evolving treatment landscape for this glomerular disease.

## 2 Clinical presentation and indications for kidney biopsy

As we have observed in our clinical practices, patients with IgAN exhibit a wide spectrum of presentations, ranging from asymptomatic cases to progressive kidney disease (Figure 1) (1, 3, 11, 12). A common IgAN presentation in pediatric patients and young adults is recurrent episodic macroscopic hematuria with concurrent upper respiratory tract infection (“synpharyngitic” hematuria) or gastrointestinal tract illness, with or without proteinuria between episodes of hematuria (8, 11–13). In comparison, adults with IgAN frequently present with asymptomatic microscopic hematuria, with or without proteinuria (13). In many cases, patients with IgAN are asymptomatic, with clinical signs detected incidentally on routine urinalysis (11, 13). Importantly, hematuria and proteinuria are common clinical signs of IgAN, but they can also indicate a wide range of glomerular diseases (13–15). IgAN may be more likely when these signs are noted in combination with certain clinical patterns, like synpharyngitic hematuria (8, 13). Additionally, while most cases of IgAN are sporadic, a family history of IgAN should heighten clinical suspicion of the disease, as limited reports of familial clustering suggest a possible genetic predisposition in some cases (13). Furthermore, IgAN can occur secondary to many systemic conditions, including IgA vasculitis, viral infection, liver cirrhosis, inflammatory bowel diseases such as Crohn's disease and ulcerative colitis, and various autoimmune disorders such as celiac disease (1–3, 13). Secondary causes of IgAN can have distinct prognostic implications compared with primary IgAN, and effective treatment often requires addressing the underlying condition, rather than using the immunosuppressive regimens typical of IgAN management (16).

**Figure 1:**
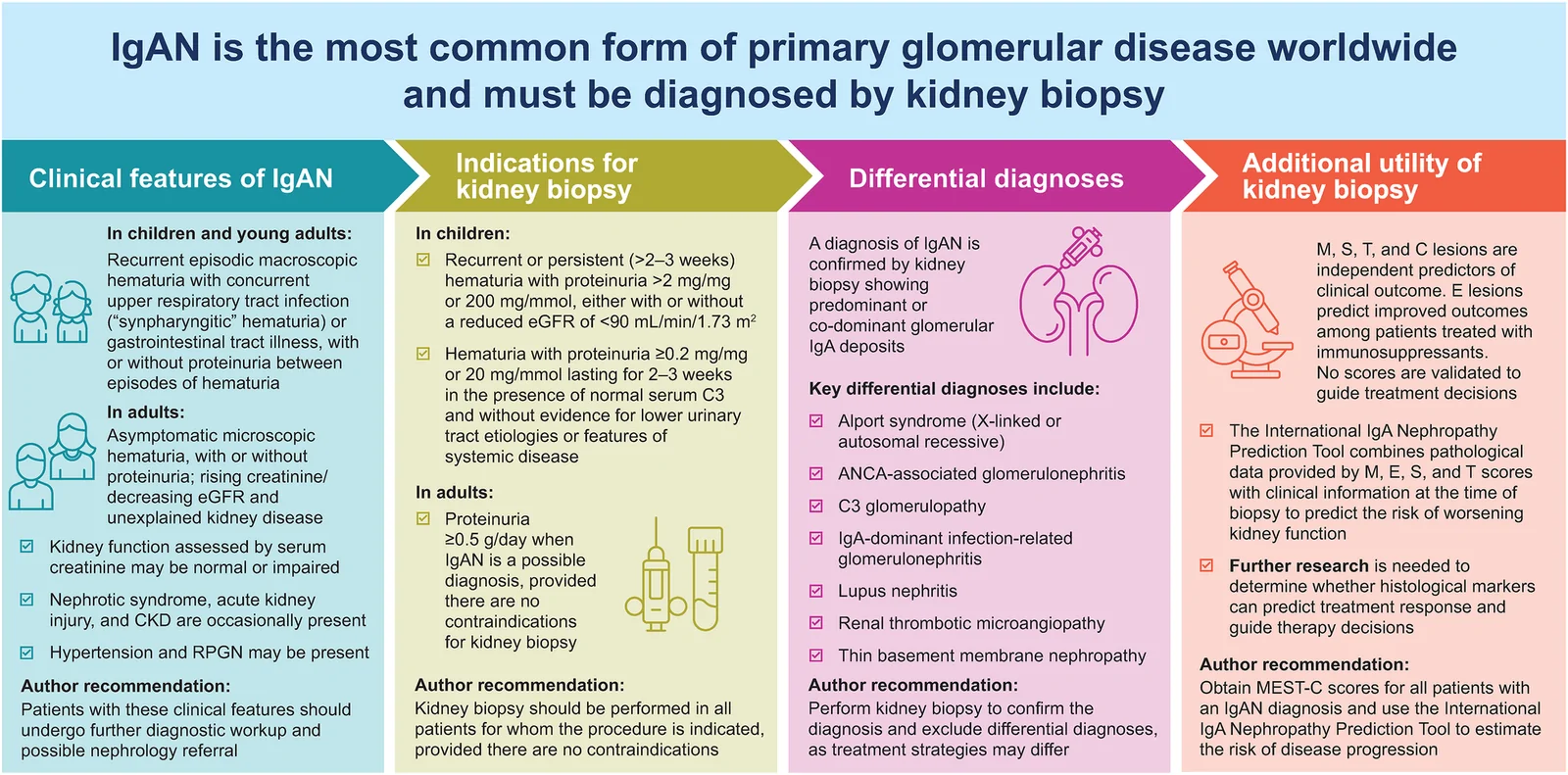
The importance of kidney biopsy in the diagnosis of IgAN, including the clinical features of IgAN that should raise clinical suspicion of the disease, the indications for kidney biopsy per clinical practice guideline recommendations, the differential diagnoses for IgAN that can be informed by kidney biopsy, and the additional prognostic utility of histological information from kidney biopsy (1, 3, 8, 13, 29, 41, 43, 44, 48). ANCA, anti-neutrophil cytoplasmic antibody; C, cellular or fibrocellular crescents; C3, complement 3; CKD, chronic kidney disease; E, endocapillary hypercellularity; eGFR, estimated glomerular filtration rate; IgA, immunoglobulin A; IgAN, immunoglobulin A nephropathy; M, mesangial hypercellularity; RPGN, rapidly progressive glomerulonephritis; S, segmental glomerulosclerosis; T, tubular atrophy/interstitial fibrosis.

Regardless of the inciting cause, there are currently no validated diagnostic serum or urine biomarkers for IgAN, representing a significant unmet need (3). Due to this lack of specific clinical signs and validated diagnostic biomarkers, kidney biopsy remains the gold standard for establishing a definitive diagnosis, as recommended by both the Kidney Disease: Improving Global Outcomes (KDIGO) 2025 Clinical Practice Guideline for the Management of Immunoglobulin A Nephropathy (IgAN) and Immunoglobulin A Vasculitis (IgAV) (3) and the International Pediatric Nephrology Association (IPNA) Clinical Practice Recommendations for the Diagnosis and Management of Children with IgAN (Figure 1) (8). The KDIGO 2025 Clinical Practice Guideline recommends kidney biopsy for all adults with proteinuria ≥0.5 g/day when IgAN is a possible diagnosis, provided there are no contraindications for the procedure (3). In children, the IPNA strongly recommends kidney biopsy for recurrent or persistent (>2–3 weeks) hematuria and nephrotic-range proteinuria (>2 mg/mg or 200 mg/mmol), either with or without a reduced estimated glomerular filtration rate (eGFR) of <90 mL/min/1.73 m^2^ (8). The IPNA also strongly recommends kidney biopsy in patients with microscopic and/or macroscopic hematuria and proteinuria ≥0.2 mg/mg or 20 mg/mmol lasting for 2–3 weeks, confirmed in ≥2 measurements taken 1–2 weeks apart, when serum complement 3 (C3) levels are normal and there is no evidence for lower urinary tract etiologies or features of systemic disease (8).

Despite the advantages of kidney biopsy and its recommendation in current guidelines, there are contraindications and potential risks associated with the procedure that must be considered (3, 4, 17–27). A kidney biopsy is an invasive procedure that should be performed when a definitive diagnosis is likely to inform treatment decisions or disease progression and prognosis, and should be avoided when the potential risks outweigh the expected clinical benefits (23). Important contraindications to kidney biopsy include factors that increase the risk of post-procedure bleeding, such as coagulation disorders, higher-grade anemia, and the use of anticoagulation agents, which should be withheld for a sufficient time to allow reversal of the anticoagulant effect before and after the biopsy, if safe to do so (17, 23). In patients with uncontrolled hypertension, kidney biopsy should be delayed until the hypertension is adequately controlled (17, 23). A kidney biopsy should also be avoided in the presence of infection affecting either the kidney or the skin over the needle site, both of which can increase the risk of sepsis (23). Lastly, in patients with cognitive impairment who are unable to cooperate with the biopsy or follow instructions, the risk of injury during the procedure may exceed the likely benefits (17, 23). Nevertheless, kidney biopsies can generally be performed without complications when contraindications are considered and appropriately managed (17).

## 3 Real-world utilization and barriers to kidney biopsy

The worldwide prevalence of IgAN varies widely by region and ethnicity (11). Among all kidney biopsy diagnoses, IgAN is most common in populations from Asia (up to 69%), followed by those in Europe (up to 53%), Oceania (up to 34%), the Americas (up to 27%), and Africa (up to 18%) (28). Although IgAN is one of the most common disorders found on kidney biopsy, real-world data related to kidney biopsy practices in IgAN specifically are limited. Regional and ethnic differences in IgAN incidence may be due to underlying variations in genetic background, but some heterogeneity is likely due to disparities in indications for kidney biopsy and access to screening programs, as well as practical or socioeconomic barriers (1, 11). For instance, urinary screening programs for the early detection of kidney diseases are widely implemented in Asian countries such as Japan, Korea, Taiwan, and Singapore, and may contribute to the higher reported incidences of IgAN in this region (11). As Japan's screening program has also been linked to a decreased rate of kidney failure (11), global implementation of such programs should be considered, to facilitate earlier detection, histological diagnosis, and treatment of IgAN.

Patients with IgAN often experience delays between the appearance of their first clinical signs and receipt of a confirmed diagnosis. In a large, retrospective, US cohort of patients with IgAN, mean serum creatinine and eGFR at time of diagnostic biopsy among 3,828 patients with available data were 3.0 mg/dL and 44.2 mL/min/1.73 m^2^, respectively. Overall, 75% of these patients presented at CKD stage ≥3, and mean proteinuria among 2,280 patients with available data was 4.2 g/day, with values ≥1 g/day observed in 85% of patients (29). Such signs of severe kidney impairment at diagnosis suggest that these patients were referred late for kidney biopsy (29, 30).

Diagnostic delays for patients with IgAN may occur with initial primary care provider (PCP) or urologist consultation, which slow subsequent nephrology referral (31, 32). Notably, a study conducted in the United States found that only 45% of patients with eGFR <30 mL/min/1.73 m^2^ in whom significant and often irreversible kidney damage had occurred were referred to nephrology by their PCPs (33). These findings underscore the need for earlier referral, ideally well before advanced kidney disease develops. Slow PCP referral to nephrology of patients with advanced kidney disease may be influenced by many factors, such as challenges in recognizing the clinical signs or severity of disease, unfamiliarity with the appropriate clinical guidelines or diagnostic workup, the perception that medical management does not improve patient outcomes, poor communication or collaboration between PCPs and nephrologists, and the unclear roles of each provider type in disease management (33, 34). Therefore, it is important to raise awareness of kidney disease diagnosis and management among PCPs, and PCPs and nephrologists must work together to promote timely referral. Avoiding diagnostic delays is increasingly crucial in light of the numerous therapies that have recently been approved for adults with IgAN, which may well improve patient outcomes in the upcoming years (35).

Healthcare system challenges may also impede biopsy in IgAN. For example, kidney biopsies may be underutilized in many regions due to a shortage of nephrologists and/or specialized renal pathologists, or limited infrastructure for specialized techniques such as immunofluorescence and electron microscopy to distinguish IgAN from other histological diagnoses (25). In areas without a centralized healthcare system, such as the United States, socioeconomically deprived individuals may be less likely to access healthcare resources (6).

In line with our clinical experience, real-world studies have demonstrated the utility and safety of kidney biopsy for kidney disorders, and complications are usually uncommon, mild when occurring, and typically do not require intervention with experienced physicians (17). However, access to kidney biopsy may be complicated by factors such as the invasive nature of the procedure, the need for expertise and ultrasound guidance for safe completion, the requirement for sedation or anesthesia, contraindications including clotting disorders or active infection, and potential complications such as postoperative bleeding and pain (17). To address these challenges and improve disease understanding, diagnosis, and treatment, the ongoing Kidney Precision Medicine Project, led by the United States, will serve as a valuable future resource for data on kidney biopsy-related complications, the clinical utility of histopathological findings, and patient-reported kidney biopsy outcomes among patients with CKD, including IgAN (36).

## 4 Diagnostic and prognostic significance of kidney biopsy

It is important to balance the risks and benefits of kidney biopsy for individual patients, but in those without contraindications, kidney biopsy should be performed as early as possible to confirm diagnosis, gain information about disease severity, and inform shared decision-making with patients.

### 4.1 Assessing differential diagnoses and establishing a specific diagnosis

Kidney biopsy is essential to distinguish IgAN from other glomerular diseases with overlapping clinical presentations (Table 1; Figure 1) (1–3, 8, 10–13, 15, 37–42). Important differential diagnoses for IgAN include thin basement membrane nephropathy, IgA-dominant infection-related glomerulonephritis, C3 glomerulopathy, and Alport syndrome. The latter is especially important in male patients with a family history of CKD and concomitant hearing loss; genetic testing should be considered in such cases (8, 37).

**Table 1:** Clinical presentations and key kidney biopsy findings for IgAN and differential diagnoses.

Diagnosis	Etiology	Clinical presentations	Kidney histological features
IgAN	Secretion of galactose-deficient IgA1 by B cells with abnormal *O*-glycan biosynthesis followed by formation of circulating polymeric immune complexes deposited in the glomerular mesangium. May occur idiopathically or secondary to IgA vasculitis, viral infection, inflammatory bowel disease, autoimmune disease, liver cirrhosis, or IgA-dominant infection-related glomerulonephritis (1–3)	Highly variable/non-specific and often asymptomatic (1, 13) In adults, asymptomatic microscopic hematuria with or without concomitant proteinuria (1, 13) In children and young adults, recurrent episodic macroscopic hematuria with concurrent upper respiratory tract infection (“synpharyngitic” hematuria) or gastrointestinal tract illness, with or without proteinuria between episodes of hematuria (8, 11–13) In children, severe hematuria and/or nephrotic-range proteinuria ≥3.5 g/day (12) Nephrotic syndrome, acute kidney injury, and CKD are occasionally present (1, 8, 13) Hypertension and RPGN may also be present (1, 8, 13)	Dominant or co-dominant mesangial deposits of polymeric IgA, predominantly of the IgA1 subclass, on routine immunofluorescence or immunoperoxidase microscopy (10) Subendothelial IgA deposits may also be detected in the glomerular capillary wall (10)
Alport syndrome (X-linked or autosomal recessive)	Mutations in *COL4A3*, *COL4A4*, and *COL4A5* genes resulting in defective assembly of the *α*3, α4, and α5 heterotrimer of Type IV collagen, which is an essential component of basement membranes in the glomeruli, cochlea, and ocular lens (37)	Microscopic hematuria (37) Flares of albuminuria with macroscopic hematuria (37) Ocular abnormalities (corneal opacities, anterior lenticonus, cataracts, retinal abnormalities) (37) Sensorineural hearing loss (37) Positive family history for CKD and hearing loss, particularly in individuals of male sex (8, 37)	In early stages, thinning and splitting of the glomerular basement membrane is observed on electron microscopy, progressing to severe splitting and irregular membrane thinning and thickening with a characteristic “basket-weave” pattern (37) In advanced stages, glomerulosclerosis and podocyte foot process effacement may also be present (37)
ANCA-associated (pauci-immune) glomerulonephritis	Activation of neutrophils by ANCAs raised against neutrophil proteins PR3 or MPO in and around microvasculature, leading to tissue damage and endothelial injury mediated by effector T cells and leukocytes (38)	Elevated serum creatinine with variable degrees of proteinuria (38) Symptoms of systemic vasculitis (weight loss, malaise, fatigue, arthralgia, myalgia) (38) May also be accompanied by signs and symptoms of small vessel vasculitis in the ear, nose, throat, eyes, airways, lungs, peripheral nervous system, skin, heart, and gastrointestinal tract (38)	Segmental necrosis of glomerular capillary loops with minimal Ig or complement deposits (38) In the acute phase, segmental necrosis, fibrin and erythrocyte extravasation into the urinary space, cellular crescents, and destruction of the glomerular basement membrane may be present, leading to global or segmental glomerulosclerosis (38) Inflammation of small arteries and a variable interstitial infiltrate around necrotic lesions in a granuloma-like pattern are common (38)
C3 glomerulopathy	Glomerular deposition of C3 driven by overactivation of the alternative complement pathway, often due to genetic and/or acquired defects in alternative pathway regulatory proteins (39)	Often indolent; may also present as a rapidly progressive glomerulonephritis (39) Hematuria and proteinuria (40) Upper respiratory tract infection, often in children and young adults (40) Acquired partial lipodystrophy (39) Retinal drusen (39)	Dominant C3 staining with intensity ≥2 orders of magnitude greater than any other immunoreactant on immunofluorescence microscopy (39)
IgA-dominant infection-related glomerulonephritis	Immune-complex mediated glomerulonephritis associated with response to bacterial infection, including methicillin-resistant and methicillin-sensitive *Staphylococcus aureus*, *Escherichia coli*, *Staphylococcus epidermidis*, and *Klebsiella* (15)	Bacteremia (15) Skin and joint infection (15) Pneumonia (15) Osteomyelitis (15) Endocarditis (15)	Endocapillary proliferation with prominent neutrophil infiltration is common. Isolated mesangial glomerulonephritis or crescentic glomerulonephritis may be present (15) Co-dominant mesangial deposits of IgA and C3, with dominant expression of kappa light chain over lambda light chain on immunofluorescence microscopy (15) Electron-dense mesangial and capillary wall deposits on electron microscopy; subepithelial humps and subendothelial deposits may also be present (15)
Lupus nephritis	Inflammation of the kidneys as a result of systemic lupus erythematosus (15)	Often asymptomatic (15) Proteinuria ≥0.5 g/day (15)	Endocapillary hypercellularity, neutrophil infiltrate, karyorrhexis, fibrinoid necrosis, hyaline deposits, cellular or fibrocellular crescents, interstitial inflammation or fibrosis, global and segmental glomerulosclerosis, and tubular atrophy (15) Podocyte foot process effacement, collapsing glomerulopathy, and vascular lesions may also be present (15)
Renal thrombotic microangiopathy	Endothelial injury leading to microvascular thrombosis occurring secondary to a wide range of conditions, including infection, drug use, malignancy, and autoimmune disease. Primary forms may be driven by *ADAMTS13* deficiency, pathogenic gene variants in coagulation proteins, or overactivation of the alternative complement pathway (41)	Microangiopathic hemolytic anemia (anemia, thrombocytopenia, reticulocytosis, elevated lactate dehydrogenase and bilirubin, low haptoglobin schistocytes) (41) Hematuria and proteinuria (41) Hypertension (41)	Glomerular or arteriolar microthrombi, arterial intimal edema, and arterial intimal fibrin (41) Red blood cell fragments and glomerular endotheliosis or mesangiolysis may also be present (41) Chronic changes include double contouring of the glomerular capillary wall and arterial onion skinning (41)
Thin basement membrane nephropathy	Mutations in *COL4A3* and *COL4A4* genes resulting in defective assembly of the α3 and α4 chains of Type IV collagen, which is an essential component of basement membranes in the glomeruli, cochlea, and ocular lens (42)	Microscopic hematuria (42) Approximately 50% of cases are familial, usually with an autosomal dominant pattern of inheritance (42)	Uniform thinning of the glomerular basement membrane by electron microscopy (42) No or low-intensity staining for C3, IgA, IgG, or IgM on immunofluorescence microscopy (42) Normal or mild mesangial cell proliferation on light microscopy with an increased number of small glomerular capillary lumens (42)

ANCA, anti-neutrophil cytoplasmic antibody; C3, complement 3; CKD, chronic kidney disease; IgA, immunoglobulin A; IgG, immunoglobulin G; IgM, immunoglobulin M; IgAN, immunoglobulin A nephropathy; MPO, myeloperoxidase; PR3, proteinase 3; RPGN, rapidly progressive glomerulonephritis.

Kidney biopsy may also reveal coexisting diagnoses, which is important to inform patient management. IgAN can occur in combination with conditions such as minimal change disease (13), anti-neutrophil cytoplasmic antibody (ANCA)-associated (pauci-immune) glomerulonephritis (43), thrombotic microangiopathy (44), and membranous nephropathy (45). Patients with IgAN and acute kidney injury may have concomitant ANCA-associated glomerulonephritis, and those experiencing nephrotic syndrome (proteinuria >3.5 g/day, edema, and hypoalbuminemia) may have concurrent minimal change disease (3).

### 4.2 Prognostic value of kidney biopsy in patients with IgAN

As demonstrated in several observational studies, early IgAN detection and intervention are of importance for best patient outcomes. Evaluation of the Validation Study of the Oxford Classification of IgAN (VALIGA) cohort (a retrospective study including 1,147 patients with IgAN across 13 European countries) demonstrated that a substantial proportion (26%) of patients experience kidney failure or 50% reduction in eGFR within 10 years of diagnosis (18). Highlighting the negative impact of diagnostic delays, a retrospective study by the United Kingdom's National Registry of Rare Kidney Diseases (RaDaR) reported baseline CKD stage as the strongest predictor of 10-year kidney survival in patients with IgAN, and specified that a sustained annual eGFR decline of 3 mL/min/1.73 m^2^ would result in all patients <40 years of age reaching ESKD within their expected lifetimes (4). Furthermore, a retrospective Norwegian study with a median follow-up of 16 years reported that risk of progression to ESKD or death was significantly associated with CKD stage and proteinuria at the time of IgAN diagnosis (20). Similarly, a retrospective analysis conducted in the United States found that lower baseline eGFR and higher baseline proteinuria (≥0.5 g/g) were significantly associated with a higher risk of ≥50% eGFR decline, kidney failure, or death within a median follow-up of 3.1 years (19). However, a retrospective Korean study of 153 patients with IgAN who had relatively benign clinical presentation at diagnosis (eGFR ≥60 mL/min/1.73 m^2^ and proteinuria <0.5 g/day) showed that over a median follow-up of 95 months, 31% of patients achieved clinical remission but 4% reached kidney failure (46). This study highlights that despite patients being diagnosed early, some still progress to kidney failure (46).

If diagnosed early, children with IgAN generally exhibit superior prognosis and disease outcomes compared with adults (4, 12, 47). Hematuria and proteinuria are typically more severe in children versus adults (12); thus, the presence of macroscopic hematuria in children may contribute to their improved prognosis by prompting faster nephrology referral (12, 47). Importantly, because even mild hematuria and proteinuria can indicate underlying glomerular disease, PCPs and nephrologists must consider whether kidney biopsy is indicated in all such cases to facilitate an early diagnosis.

Beyond the direct benefits afforded by a timely IgAN diagnosis, kidney biopsy findings can inform the extent of disease activity and predict outcomes (Figure 1). The MEST-C score, as defined in the Oxford Classification of IgA Nephropathy, is widely used to assess kidney biopsies. It scores the severity of five pathological parameters: mesangial hypercellularity (M), endocapillary hypercellularity (E), segmental glomerulosclerosis (S), tubular atrophy/interstitial fibrosis (T), and cellular or fibrocellular crescents (C) (48, 49). M, S, T, and C scores are independent predictors of clinical outcome, of which the T score is the most robust, while E lesions predict improved outcomes among patients treated with immunosuppressants (48). For both adults and children, the International IgA Nephropathy Prediction Tool combines pathological data (M, E, S, and T scores) with clinical information collected up to two years from the time of biopsy to predict the risk of worsening kidney function within the subsequent seven years (3, 21, 22). While these tools serve as valuable resources to quantify disease progression risk and inform shared decision-making with patients, neither has been validated to help guide treatment decisions in IgAN (3, 48).

## 5 Discussion

### 5.1 Future implications for patient management and therapy

Historically, IgAN-treating providers have focused on optimizing supportive care, including blood pressure management, renin–angiotensin system inhibitors, lifestyle changes, and interventions to reduce cardiovascular risk (15). However, the therapeutic landscape for IgAN is rapidly evolving, as demonstrated by the recent US Food and Drug Administration approvals of delayed-release budesonide, sparsentan, iptacopan, atrasentan, and sibeprenlimab for the treatment of adults with primary IgAN at risk of disease progression, with additional promising treatment strategies under investigation (2, 50–52).

Despite the necessity of kidney biopsy for the diagnosis of IgAN, there is currently no classification system providing treatment recommendations based on biopsy findings (3). Observational data suggest that patients with E or C lesions may benefit from corticosteroid therapy (48), but MEST-C has not been validated for treatment decision-making, and current guidelines do not recommend immunosuppression based on MEST-C score (3, 48).

With the increasing availability of targeted IgAN therapies, and considering the limitations of MEST-C and the lack of established clinical biomarkers to guide treatment, further research is needed to determine whether histological markers can predict treatment response and guide therapy decisions. Additionally, the establishment of an updated histological classification system will be essential to support precision-based treatment approaches. Future research should also focus on the development of validated noninvasive biomarkers. For example, in IgAN, elevated serum IgA/C3 ratio, high serum and urinary Gd-IgA1, and urinary C4d and CD163 have been associated with poor kidney outcomes (53–56). Disease-specific biomarkers could improve diagnostic accessibility including earlier diagnosis, support longitudinal disease monitoring, and provide alternatives for patients for whom kidney biopsy is contraindicated (57, 58). Furthermore, biomarkers could also be coupled with histologic data, where available, to further stratify patients according to their biological characteristics to help inform treatment selection and monitor treatment effect (58). However, these approaches remain under investigation and are not yet validated for routine clinical use (3).

Repeat kidney biopsies can detect changes in pathological features due to disease progression or response to treatment (3, 48). However, recommending this procedure in practice requires careful risk–benefit analysis for each patient. The KDIGO 2025 Clinical Practice Guideline recommends repeat kidney biopsy only for IgAN with acute kidney injury, when kidney function does not improve within two weeks of cessation of hematuria (3). For children, the IPNA suggests repeat kidney biopsy to validate initial biopsy findings in the context of relapsed IgAN (i.e., a presentation of worsening proteinuria with or without increased microhematuria, and/or an unexpected eGFR decline) (8). There is a paucity of data on the correlation between treatment responses and longitudinal biopsy findings, with clinical trials often allowing patients to enroll despite their lack of recent kidney biopsy. Importantly, in patients with evidence of advanced fibrotic disease, a repeat biopsy is unlikely to provide additional actionable information. As such, the potential risk of procedural complications may exceed the likely benefits, warranting careful assessment on a case-by-case basis (23). Further research is needed to validate the utility of repeat biopsy in native kidneys with IgAN and to determine its usefulness for guiding individualized treatment strategies (48).

Despite its necessity, kidney biopsy also has inherent limitations. First, it is subject to sampling error; only a small portion of kidney tissue is examined, which may result in an unrepresentative assessment of disease pathology as lesions may be heterogeneously distributed, potentially leading to misleading diagnostic and prognostic information (59–61). Second, MEST-C scores represent a single time point and may not adequately reflect ongoing disease activity or dynamic changes in response to therapy (3). Biopsy findings and MEST-C scores should therefore be interpreted alongside clinical parameters when assessing prognosis and guiding treatment decisions (3). Third, interobserver variability in biopsy interpretation may also affect its prognostic value, with the scoring of E and C lesions in MEST-C showing particularly poor reproducibility (62). However, enhanced education and the development of automated scoring approaches may help improve reproducibility and mitigate these limitations (62, 63). Finally, patient perceptions, such as fear, uncertainty, and limited understanding of the procedure, can act as barriers to kidney biopsy (24). Therefore, it is important that efforts are made to reduce these barriers by providing clear patient education and increased trust between patients and their healthcare providers (24).

### 5.2 Conclusion

Although a diagnosis of IgAN may be suspected based on clinical findings, it can only be definitively established through kidney biopsy (1, 3, 8, 13, 29, 41, 43, 44, 48). Additionally, the histological data obtained on biopsy are of clinical value for predicting patient outcomes and informing shared decision-making (3, 21, 22, 48). Thus, we strongly recommend that kidney biopsy be completed in all patients with suspected IgAN who meet guideline-recommended criteria, with no procedural contraindications, including adults with proteinuria ≥0.5 g/day and children with persistent/recurrent hematuria and nephrotic-range proteinuria (>2 mg/mg or 200 mg/mmol), with or without eGFR <90 mL/min/1.73 m^2^. We acknowledge that there are multiple barriers preventing the global implementation of this procedure. However, we hope that this perspective will improve provider education about IgAN and emphasize the importance of diagnostic biopsy as a means to increase patient identification and promote timely nephrology referral. Ultimately, this will allow earlier implementation of therapy to improve outcomes in this patient population.

## Data Availability

The original contributions presented in the study are included in the article. Further inquiries can be directed to the corresponding author.
